# Robotic Rectopexy: A Single-Centre Experience

**DOI:** 10.7759/cureus.98398

**Published:** 2025-12-03

**Authors:** Anil Kumar, Raheel Anis, Sajal Rai, Maria Javid, Madan Palliyil

**Affiliations:** 1 Colorectal and General Surgery, Stepping Hill Hospital, Stockport NHS Foundation Trust, Stockport, GBR; 2 Colorectal and General Surgery, United Lincolnshire Hospital NHS Trust, Boston, GBR; 3 General Surgery, Stepping Hill Hospital, Stockport NHS Foundation Trust, Stockport, GBR

**Keywords:** intracorporeal, laparoscopy, rectal prolapse, rectopexy, robotic

## Abstract

Background and aims

Rectal prolapse and rectocele are socially and psychologically debilitating conditions. While laparoscopic rectopexy has improved outcomes over open surgery, it faces limitations in pelvic access, mesh positioning, and suturing. Robotic-assisted rectopexy may overcome these challenges through enhanced visualisation and manoeuvrability. This study presents a single-centre experience of robotic rectopexy.

Methods

A retrospective review was conducted at Stepping Hill Hospital, Stockport, UK, from 2019-2024. Data collected included operative time, intra- and postoperative complications, analgesia requirement and length of stay.

Results

Twenty-one patients underwent robotic rectopexy. The mean operative time was three hours and 18 minutes. No conversions or intraoperative complications were observed. All patients were managed with simple analgesia. Fifteen (71.4%) were discharged on post-operative day (POD) 1, four patients on POD 2 and two patients were discharged later (POD >3). The mean hospital stay was 1.6 days. Two patients had small postoperative collections managed conservatively. One recurrence was observed on follow-up.

Conclusions

Robotic rectopexy is a safe and effective technique for rectal prolapse, demonstrating minimal complications and short hospital stays. Further research is warranted to assess long-term outcomes.

## Introduction

Rectal prolapse is a debilitating condition characterized by circumferential intussusception of the rectal wall through the anal canal, leading to distressing symptoms such as fecal incontinence, mucus discharge, bleeding, and a sensation of incomplete evacuation. Rectocele, commonly occurring in female patients, involves bulging of the rectal wall into the posterior vaginal wall, often contributing to obstructed defecation. Both conditions significantly impair quality of life and carry social and psychological burdens.

Abdominal rectopexy for complete rectal prolapse was first described by Ripstein in 1952 [1]. Since then, numerous modifications have been introduced, aimed at improving anatomical restoration while minimizing recurrence and complications. Among the various surgical techniques, minimally invasive ventral rectopexy has become a widely adopted procedure for the management of rectal prolapse, offering favorable outcomes with lower morbidity compared to traditional posterior approaches.

While laparoscopic ventral rectopexy offers benefits such as reduced postoperative pain and faster recovery, it presents challenges in deep pelvic dissections, particularly with mesh positioning and intracorporeal suturing. These technical limitations are more pronounced in patients with prior pelvic surgeries or complex anatomy.

The robotic surgical platform addresses many of these issues by providing high-definition three-dimensional visualization, wristed instruments for enhanced dexterity, and improved ergonomics. As a result, robotic rectopexy has gained attention as a viable alternative, particularly in complex or recurrent cases.

Despite growing adoption, real-world data on robotic rectopexy remains limited, particularly from single-institution experiences. This study aims to evaluate the perioperative outcomes, postoperative recovery, and complication rates associated with robotic rectopexy at a single center in the United Kingdom.

## Materials and methods

Study design and setting

This retrospective, single-center observational study was conducted at Stepping Hill Hospital, Stockport, United Kingdom. The study included all patients who underwent robotic rectopexy for full-thickness rectal prolapse or rectocele between January 2019 and December 2024. All procedures were performed by a single colorectal surgeon, and the median duration of postoperative follow-up in the outpatient clinic was two years

Patient selection

Patients were included if they underwent robotic ventral rectopexy using the da Vinci® surgical system (Intuitive Surgical, Inc., Sunnyvale, CA, USA). Exclusions were combined procedures, incomplete records, or conversion to open/laparoscopic approaches.

Data collection

Data extracted included demographics, operative time, intraoperative complications, conversion rate, postoperative analgesia, length of stay, postoperative complications, and recurrence during follow-up.

Surgical technique

Robotic-assisted ventral mesh rectopexy was performed under general anesthesia. Pneumoperitoneum was established, the robot docked, and the anterior dissection of the rectovaginal/rectovesical plane carried out to the levator ani. Polypropylene mesh was secured to the anterior rectal wall and fixed to the sacral promontory. The peritoneum was closed over the mesh.

Outcome measures

Primary outcomes were operative time, length of stay, postoperative complications, and recurrence.

Statistical analysis

Descriptive statistics were used; continuous variables as mean ± SD, categorical as frequency and percentages.

## Results

A total of 21 patients underwent robotic rectopexy at Stepping Hill Hospital between 2019 and 2024.

Operative outcomes

The mean duration of surgery was three hours and 18 minutes (198 minutes). There were no intraoperative complications and no conversions to either conventional laparoscopic or open approaches. The robotic approach allowed for complete anterior dissection and mesh placement in all patients without technical difficulty.

Postoperative recovery

**Figure 1:**
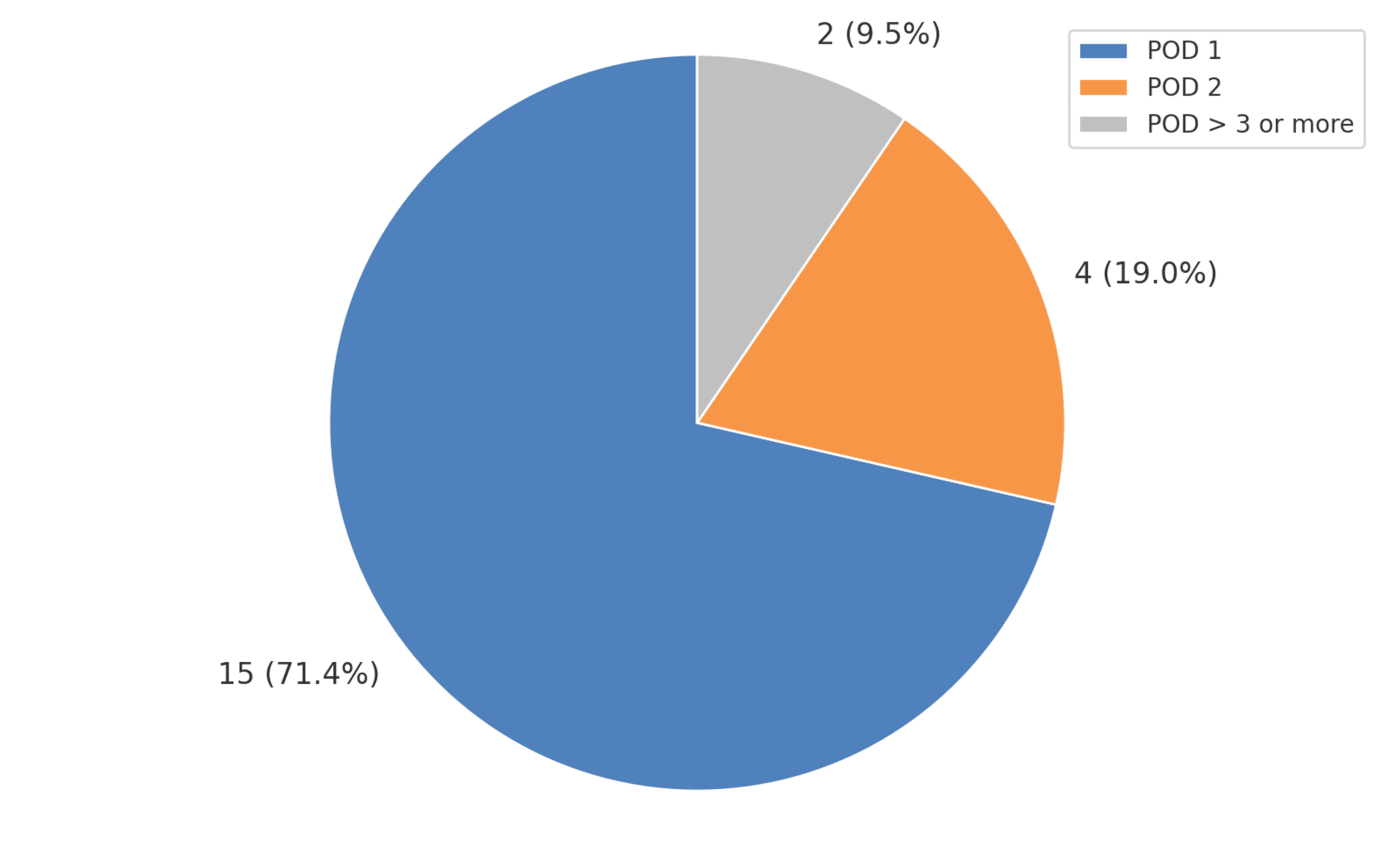
Pie chart illustrating the post-operative discharge distribution for total 21 patients undergoing robotic rectopexy POD: post-operative day

All patients were managed with simple oral analgesia postoperatively, with no requirement for intravenous opioids or escalation. The majority of patients, 15 (71.4%), were discharged on postoperative day (POD) 1, while four patients (19%) were discharged on POD 2. The remaining two were discharged on POD >3 or more. The mean length of hospital stay was 1.6 days. As shown in the pie chart, postoperative discharge days are shown in Figure 1.

Postoperative complications

**Figure 2:**
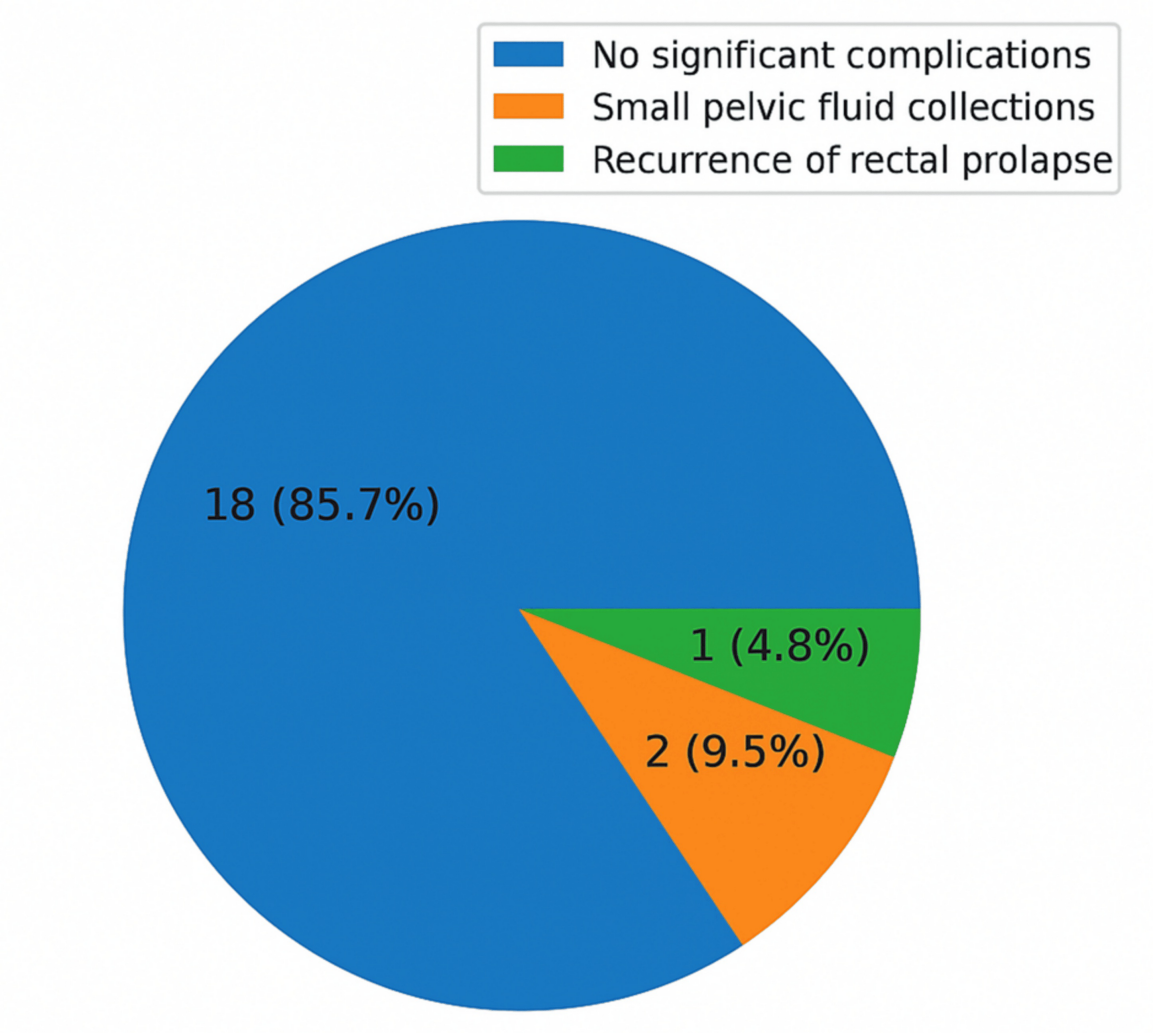
Pie chart representation of postoperative complications

Among 21 patients, 18 (85.7%) experienced no significant postoperative complications. Two patients developed small pelvic fluid collections during the immediate postoperative period, identified on ultrasound imaging. These were managed conservatively with observation and oral antibiotics, without the need for drainage. There were no readmissions, no wound infections, and no anastomotic complications. At a median follow-up, one patient (4.8%) was found to have clinical recurrence of rectal prolapse. This case is under further evaluation and consideration for re-intervention. Figure 2 below shows a graphic representation of postoperative complications.

## Discussion

Robotic rectopexy has emerged as a significant advancement in minimally invasive rectopexy, particularly in addressing the technical challenges inherent to conventional laparoscopic surgery in the deep pelvis. The narrow working space, restricted range of motion of straight laparoscopic instruments, and demanding intracorporeal suturing can limit operative efficiency and precision [2]. Robotic platforms, equipped with articulated instruments, tremor filtration, and three-dimensional (3D) high-definition visualisation, are designed to overcome these constraints and may enhance dissection accuracy, nerve preservation, and mesh fixation [3,4]. Several studies have confirmed these advantages, reporting improved ergonomics and surgeon comfort during technically demanding procedures [5].

Our series of 21 robotic rectopexy procedures highlights the feasibility, safety, efficiency and technical reliability of this minimally invasive approach, with perioperative outcomes aligning with and in some respects surpassing those reported for laparoscopic ventral rectopexy [6]. The mean operative time of 198 minutes in our cohort is slightly longer than the 150-190 minutes typically reported in laparoscopic surgery [7]. We attribute this difference primarily to the initial learning curve associated with setting up the robotic system, docking, and adapting to new ergonomics. This finding is consistent with prior studies, which have demonstrated that early robotic series had longer operative times compared to laparoscopy, but with a progressive reduction as institutional and surgeon experience increased. Hashimoto et al. and Trilling et al. [8,9], for example, reported that with accumulated experience, artificial intelligence, machine learning and robotic surgery can approach or even equal laparoscopic benchmarks.

Importantly, no intraoperative complications or conversions to open surgery occurred in our study, highlighting both the safety profile and technical stability of the robotic platform in complex pelvic dissections. Similar findings have been reported in other robotic series, including those by Trilling et al. [9], reinforcing the low risk of conversion when robotics is employed in deep pelvic procedures [10].

In our cohort, we observed a swift post-operative recovery. The majority of our patients, 15 (71%), were discharged on POD 1 with only regular analgesics. The average hospital stay was just 1.6 days, which is far shorter than the historical duration of stay of three to six days following conventional rectopexy [10,11]. Our findings are in keeping with available literature on robotic rectopexies, which offers the benefits of reduced postoperative pain and shorter hospitalisation when compared to open rectopexy. These outcomes parallel those reported in ambulatory robotic ventral rectopexy, where selected patients were safely discharged within 24 hours. Similar findings were reported in a meta-analysis conducted by Bao et al. in 2021, where researchers found reduced length of stay and faster return to function following robotic ventral mesh rectopexy compared to laparoscopic techniques [12].

The overall complication rate in our study was low (9.5%) and comprised minor pelvic collections, which were managed conservatively without the need for any intervention. This compares favorably with morbidity rates reported in published robotic series, which typically range between 10-20%. Importantly, we observed no major complications, readmissions, or mesh-related problems, further supporting the safety profile of the robotic approach. One patient in our cohort developed recurrence, amounting to a recurrence rate of 4.8%, which aligns with the 3-10% recurrence rates reported in the literature for both robotic and laparoscopic ventral rectopexy. The long-term PEXITY study similarly demonstrated durable outcomes following laparoscopic rectopexy [5], reinforcing the principle that meticulous surgical technique is the critical determinant of recurrence. Our findings suggest that the robotic platform, with its enhanced three-dimensional visualisation and instrument articulation, may facilitate precise pelvic dissection and secure mesh fixation, thereby supporting durable outcomes.

One of the main criticisms of robotic rectopexy, and robotic surgery in general, currently, is the high procedural cost, especially in the National Health Service (NHS). The NHS has been navigating financial pressures for more than a decade, and in recent years, the financial burden has reached distressing levels. This burden has a direct implication on patient care, affecting both access and the quality of services delivered [13]. Robotic surgical systems, such as the da Vinci platform, have an acquisition cost ranging between £500,000 and £1.5 million per unit. Additionally, the expenses associated with maintenance, training, and single-use consumables further elevate the overall cost per procedure. This financial burden is particularly pronounced in low-volume centres, where the amortisation of fixed costs is less efficient, potentially impacting the cost-effectiveness of robotic interventions [14,15]. However, when we look into cost-effectiveness, we must also consider clinical and system-level benefits. The reduced pain post-operatively, shorter stay in hospital, and faster return to baseline function observed in our series translates into lower inpatient costs and potentially earlier return to work for patients. If replicated on a larger scale, such reductions in bed days could offset some of the higher upfront procedural costs, particularly in high-volume centres. In addition, the ergonomic technical advantages of the robotic system, including improved dexterity, reduced physical strain on operating surgeons, especially in prolonged surgeries, deep and difficult pelvic dissections, stable 3D visualisation, and tremor filtration, may contribute to lower complication and recurrence rates, especially in patients with challenging pelvic anatomy. While our recurrence rate (4.8%) falls within the lower end of published ranges, longer follow-up will be required to confirm durable benefit. If robotic surgery reduces re-interventions and readmissions over the long term, this could further enhance its cost-effectiveness profile. Finally, as surgical teams progress along the learning curve, operative times typically shorten, which reduces theatre occupancy costs. Our mean duration of 198 minutes reflects early experience but is expected to improve with increasing case volume. Furthermore, with more institutions investing in robotic platforms across specialities, the shared utilisation of robotic systems may distribute costs more efficiently.

In this context, while robotic rectopexy currently carries higher direct costs, the potential for indirect savings and improved patient outcomes suggests a more nuanced cost-benefit balance than raw theatre expenditure alone would imply. Despite ongoing financial constraints, the NHS has recognised the potential benefits of robotic surgery and, as part of a strategic initiative, NHS England aims to increase the number of robot-assisted surgeries to 500,000 annually by 2035, up from 70,000 in 2023/24. This expansion is anticipated to enhance patient outcomes and operational efficiency across the healthcare system [16]. Future multicentric cost-effectiveness studies incorporating not only perioperative metrics but also functional outcomes, quality-of-life data, and long-term recurrence rates are needed to define the true economic value of robotic rectopexy.

Our study is subject to several limitations. The retrospective design introduces susceptibility to selection and information bias, while the small sample size limits statistical power to detect rare complications or differences in recurrence. Furthermore, our follow-up duration was relatively short, preventing definitive conclusions regarding long-term recurrence rates, functional outcomes, or mesh-related complications. As a single-centre experience, generalisability to other institutions with differing expertise, patient populations, or healthcare resources is limited. Additionally, the potential influence of the learning curve on operative time and perioperative outcomes must be acknowledged, as has been highlighted in prior robotic series.

Despite these limitations, our findings contribute to the growing body of evidence supporting robotic rectopexy as a safe and effective treatment for rectal prolapse. Future research should focus on multicenter prospective studies with larger cohorts, extended follow-up, and standardised reporting of functional outcomes such as continence, constipation, and quality of life. Comparative cost-benefit analyses between robotic and laparoscopic approaches will also be essential to guide adoption in diverse healthcare settings.

## Conclusions

Robotic rectopexy is a safe, feasible, and effective surgical technique for managing rectal prolapse, with favorable perioperative outcomes, low complication rates, and minimal recurrence in the short term. Its technical advantages over laparoscopy facilitate precise dissection and mesh placement, contributing to improved recovery and patient outcomes. Larger prospective studies with longer follow-up are needed to validate these findings and assess long-term functional benefits.
